# Cryo-EM structure of the RADAR supramolecular anti-phage defense complex

**DOI:** 10.1016/j.cell.2023.01.012

**Published:** 2023-03-02

**Authors:** Brianna Duncan-Lowey, Nitzan Tal, Alex G. Johnson, Shaun Rawson, Megan L. Mayer, Shany Doron, Adi Millman, Sarah Melamed, Taya Fedorenko, Assaf Kacen, Alexander Brandis, Tevie Mehlman, Gil Amitai, Rotem Sorek, Philip J. Kranzusch

**Affiliations:** 1 Department of Microbiology, Harvard Medical School, Boston, MA 02115, USA; 2 Department of Cancer Immunology and Virology, Dana-Farber Cancer Institute, Boston, MA 02115, USA; 3 Department of Molecular Genetics, Weizmann Institute of Science, Rehovot, Israel; 4 Harvard Center for Cryo-Electron Microscopy, Harvard Medical School, Boston, MA 02115, USA; 5 Department of Immunology, Weizmann Institute of Science, Rehovot 7610001, Israel; 6 Life Sciences Core Facilities, Weizmann Institute of Science, Rehovot, Israel; 7 Parker Institute for Cancer Immunotherapy at Dana-Farber Cancer Institute, Boston, MA 02115, USA

**Keywords:** anti-phage immunity, phage, adenosine deaminase

## Abstract

RADAR is a two-protein bacterial defense system that was reported to defend against phage by “editing” messenger RNA. Here, we determine cryo-EM structures of the RADAR defense complex, revealing RdrA as a heptameric, two-layered AAA+ ATPase and RdrB as a dodecameric, hollow complex with twelve surface-exposed deaminase active sites. RdrA and RdrB join to form a giant assembly up to 10 MDa, with RdrA docked as a funnel over the RdrB active site. Surprisingly, our structures reveal an RdrB active site that targets mononucleotides. We show that RdrB catalyzes ATP-to-ITP conversion *in vitro* and induces the massive accumulation of inosine mononucleotides during phage infection *in vivo*, limiting phage replication. Our results define ATP mononucleotide deamination as a determinant of RADAR immunity and reveal supramolecular assembly of a nucleotide-modifying machine as a mechanism of anti-phage defense.

## Highlights

• RADAR defense system inhibits phage replication through ATP deamination
• Cryo-EM structures reveal that RdrA and RdrB assemble to form a giant 10 MDa complex
• RdrB is an adenosine deaminase that catalyzes massive accumulation of ITP and dITP
• RdrA induces RdrB activity and may control sensing of phage infection

## Graphical abstract

**Figure:**
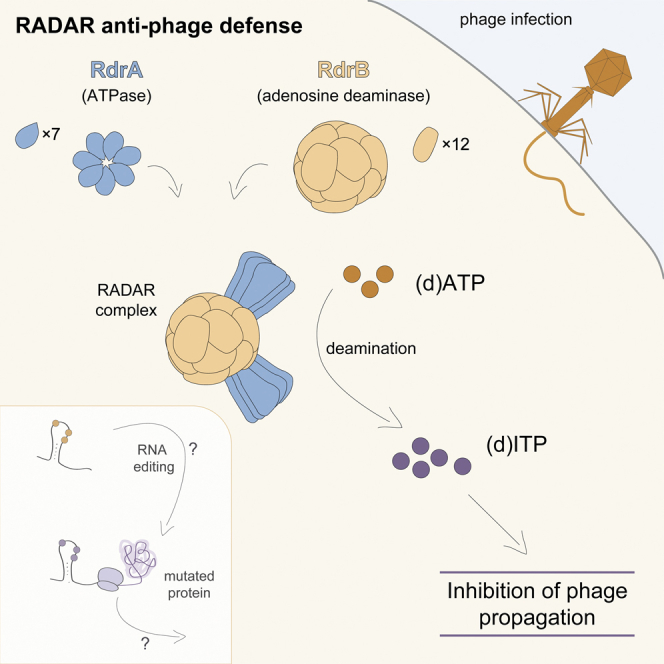

## Teaser

In the RADAR system, the adenosine deaminase RdrB forms a giant complex with RdrA, and the RADAR supramolecular complex triggers rapid ATP-to-ITP conversion in response to phage infection.

## Introduction

Bacteria encode a rich and highly diverse arsenal of anti-phage immune systems, allowing them to mitigate phage infections.^1^^,^^2^ Although most bacteria carry at least one restriction enzyme and about half of them encode CRISPR-Cas as part of their immune arsenal, other defense systems are sparsely distributed in microbial genomes.^3^^,^^4^ Over sixty anti-phage defense systems have been discovered in the past few years^5^^,^^6^^,^^7^^,^^8^^,^^9^ and although the mechanisms of action of a minority of them have been determined,^10^^,^^11^^,^^12^^,^^13^^,^^14^^,^^15^^,^^16^ the vast majority remain unexplored. It is estimated that an average bacterial genome contains more than five defense systems, with some bacteria encoding as many as 57 such systems.^4^

**Figure 1:**
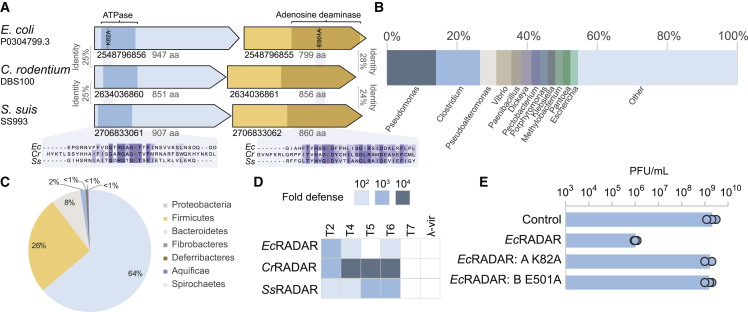
Diverse RADAR systems protect *E. coli* from phage replication (A) RADAR systems studied here. Gene IDs in the IMG database are indicated. (B) Genera of bacteria encoding RADAR. (C) Phylum distribution of RADAR-encoding bacteria. (D) RADAR systems defend against phages. RADAR systems were cloned into plasmids and transformed into *E. coli MG1655*. Fold defense was measured using serial dilution plaque assays. Data represent an average of three replicates (see detailed data in Figure S1). (E) Effect of point mutations on the defensive activity of *Ec*RADAR. Data represent plaque-forming units per mL (PFU mL^−1^) of T2 phage infecting control cells, *Ec*RADAR-expressing cells, and two strains mutated in the predicted ATPase or deaminase domains. Shown is the average of three replicates, with individual data points overlaid.

An intriguing defense system that was recently discovered is called restriction by an adenosine deaminase acting on RNA (RADAR).^6^ The RADAR system comprises two genes, *rdrA* that encodes a protein with an ATPase domain, and *rdrB*, encoding a predicted adenosine deaminase domain protein (Figure 1A). Both RdrA and RdrB are large proteins, with an average size of ∼900 amino acids each. The RADAR operon from *Citrobacter rodentium* DBS100 was heterologously expressed in *Escherichia coli* and shown to confer defense against phages T2, T3, T4, and T5, in a manner dependent on both RdrA and RdrB.^6^ The RADAR system was proposed to confer defense by editing of adenosine to inosine residues in RNA molecules of the bacterial host, thus leading to abortive infection via growth arrest or death of the infected cells.^6^

In this study, we used cryoelectron microscopy (cryo-EM) to determine the molecular structure of the RADAR defense complex. We find that RdrA forms a heptameric complex with a conserved AAA+ core and a previously uncharacterized C-terminal domain. RdrB, the adenosine deaminase protein, forms a highly atypical dodecameric shell that cages an enzymatic active site that has surprising homology to mononucleotide deaminases, not RNA-targeting enzymes. We demonstrate that the RdrA heptamer stably interacts with the RdrB dodecamer and forms a channel that feeds directly into the deaminase active site of RdrB. Multiple RdrA complexes can occupy the RdrB dodecamer, and these can together form a maximal supramolecular complex of up to 10 MDa. In contrast to previous hypotheses, we do not find strong evidence for RNA editing by the RADAR complex. Rather, we demonstrate that RADAR modifies the mononucleotides ATP and deoxy-ATP (dATP) to inosine triphosphate (ITP) and deoxy-ITP (dITP), respectively, in response to phage infection. This mononucleotide modification, which we observe both *in vitro* and *in vivo*, is suggested to block phage replication.

## Results

### Diverse RADAR systems protect *E. coli* from phage replication

A recent cross-genome defense system annotation effort detected 103 RADAR systems in ∼22,000 analyzed genomes.^4^ In the current study, we analyzed a larger set of ∼38,000 bacterial and archaeal genomes and detected RADAR in 270 of these, confirming that this defense system is rare and occurs in <1% of analyzed sequenced genomes (Table S1). Despite its rarity, the RADAR system was widely distributed phylogenetically, occurring in 75 distinct genera spanning 7 phyla in our set (Figures 1B and 1C).

We selected the RADAR systems of *E. coli* P0304799.3 and *Streptococcus suis* SS993 for further experimental investigation, as well as the previously studied *C. rodentium* DBS100 RADAR.^6^ Proteins in these systems vary substantially in amino acid sequence: RdrA proteins exhibit low overall sequence homology with significant conservation limited to only the ATPase domain that exhibits 22%–25% sequence identity (Figure 1A). RdrB amino acid sequence conservation is similarly limited, occurring in only the C-terminal portion of the protein, which harbors part of the predicted adenosine deaminase domain and exhibits 24%–28% sequence identity between distinct RADAR systems (Figure 1A). Such an extensive divergence in protein sequences is typical to proteins involved in immunity^17^ and has been observed in other rare defense proteins such as the prokaryotic viperin and gasdermin anti-phage defense operons.^10^^,^^12^ Despite the divergence in sequences, the predicted active sites were conserved in both RdrA and RdrB (Figure 1A).

**Figure S1:**
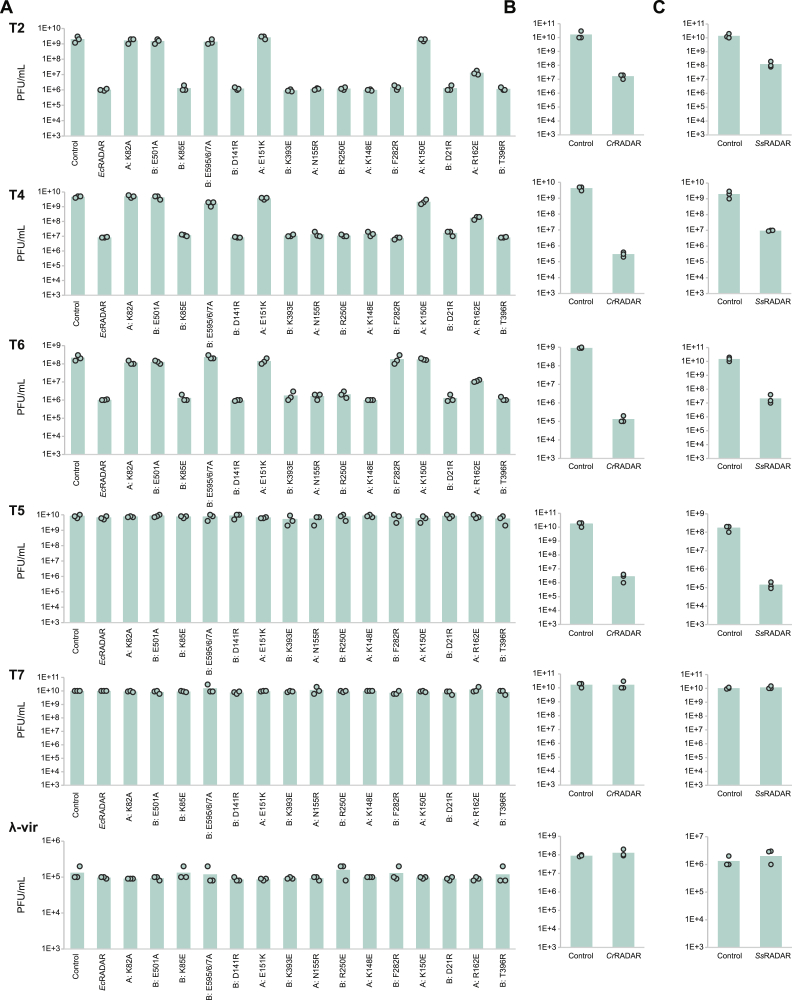
RADAR system from *E. coli* P0304799.3, *C. rodentium* DBS100, and *S. suis* SS993 protect against phage infection, related to Figure 1 (A) Bacteria expressing WT RADAR system from *E. coli* P0304799.3, as well as the system with point mutations in *rdrA* (“A”) or *rdrB* (“B”), and a negative control that contains an empty vector, were grown on agar plates in room temperature. 10-fold serial dilutions of the phage lysate were dropped on the plates. Data represent PFU mL^−1^ for phages tested in this study. Each bar graph represents average of three replicates, with individual data points overlaid. The data for T2-infected WT are also presented in Figures 1E, 3H, and 4G. (B and C) Bacteria expressing RADAR systems from *C. rodentium* DBS100 (B) and *S. suis* SS993 (C) and a negative control that contains an empty vector or a vector expressing GFP (as detailed in the STAR Methods section), were grown on agar plates in room temperature. 10-fold serial dilutions of the phage lysate were dropped on the plates. Data represent PFU mL^−1^ for phages tested in this study. Each bar graph represents average of three replicates, with individual data points overlaid.

When heterologously expressed in *E. coli* MG1655, all RADAR systems conferred defense against the closely related T-even phages T2, T4, and T6, with two of them also defending against T5, confirming the previous report on *C. rodentium* RADAR (Figures 1D and S1).^6^ Mutations in the *E. coli* RADAR, predicted to inactivate the ATPase site of RdrA or the deaminase active site of RdrB abolished defense, verifying, also in agreement with previous reports,^6^ that both enzymatic functions are necessary for defense (Figure 1E).

### Structure of RdrA reveals a two-layered heptameric ATPase assembly with a unique C-terminal domain

**Figure S2:**
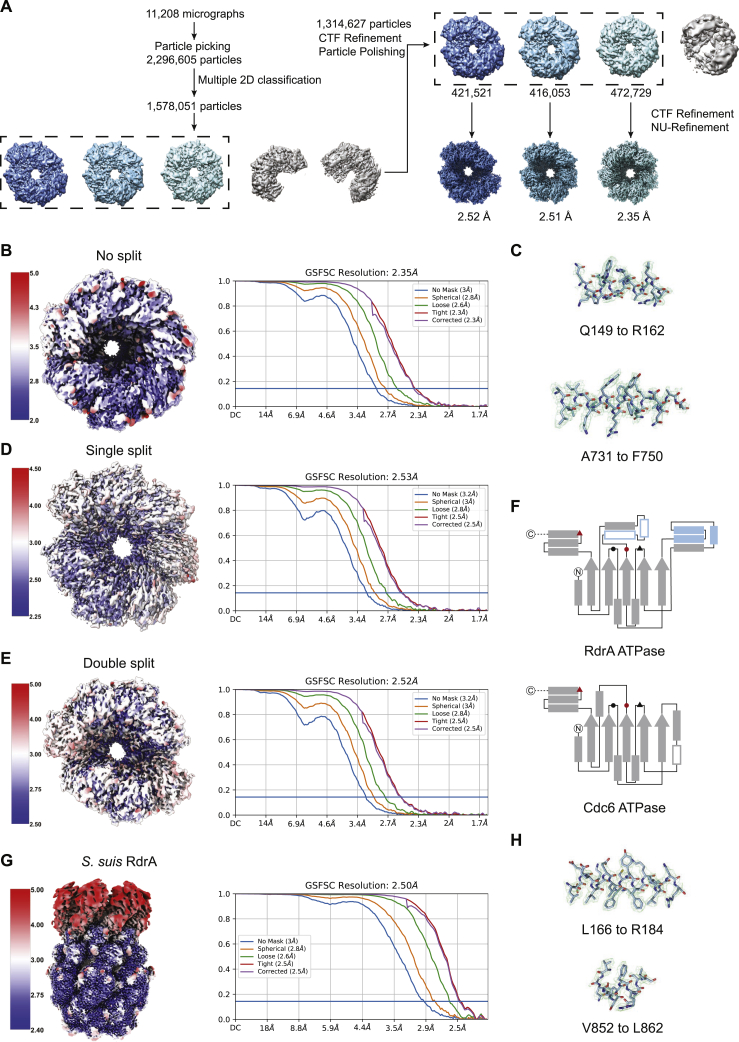
Structure of *E. coli* and *S. suis* RdrA ATPase, related to Figure 2 (A) Particle picking and classification strategy for *E. coli* RdrA. (B) Local resolution (left) and resolution by Fourier shell correlation (FSC) for unsplit *E. coli* RdrA. (C) Example model to map fit from no-split *E. coli* RdrA with the map contoured at 4.0 σ. (D and E) Local resolution (left) and resolution by FSC for *E. coli* RdrA with a single split (D) or a double split (E). (F) Cartoons comparing the ATPase domain of *E. coli* RdrA and *Drosophila* Cdc6. Conserved ATPase features are annotated as follows: Walker A/P loop (black circle), Walker B (black triangle), sensor 1 (red circle), and sensor 2 (red triangle). Insertion 1, which forms the “crown,” is shown as filled blue rectangles and insertion 2 is shown as empty rectangles outlined in blue. (G) Local resolution (left) and resolution by FSC for *S. suis* RdrA. (H) Example model-to-map fit from *S. suis* RdrA with the map contoured to 5.0 σ.

**Figure 2:**
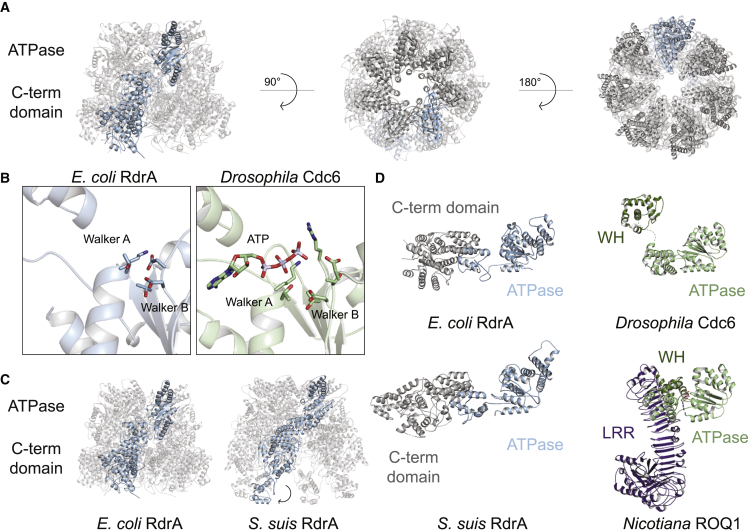
Structure of RdrA reveals a two-layered heptameric ATPase assembly with a unique C-terminal domain (A) Cartoon representation of *E. coli* RdrA cryo-EM structure. (B) Comparison of *E. coli* RdrA and *Drosophila* Cdc6 active sites, containing the well-conserved Walker A and Walker B motifs. (C) Comparison of *E. coli* RdrA and *S. suis* RdrA, highlighting the flipping out of the C-terminal helical bundle at the base of the protein. (D) Comparison of *E. coli* and *S. suis* RdrA to ATPase domain-containing proteins: *Drosophila* Cdc6 (PDB: 7JGR), part of the origin recognition complex, and *Nicotiana* ROQ1 (PDB: 7JLU, 7JLV), a plant NLR that sense the pathogen effector protein XopQ.

To define the role of RdrA in phage defense, we used cryo-EM to determine a 2.5 Å structure of *E. coli* RdrA (Figures S2A–S2C; Table S2). The cryo-EM structure of RdrA reveals a heptameric assembly with two layers of interlocking protein domains (Figure 2A). The top layer of the RdrA assembly contains seven N-terminal AAA+ ATPase domains with an active site formed between each pair of neighboring subunits. Extending from the N-terminal AAA+ domain, the RdrA C terminus is a largely α-helical lobe that interlocks around the assembly to form a flared bottom layer of the heptameric complex. We analyzed minor complexes in the *E. coli* RdrA data and determined a series of additional structures including a 2.5 Å structure of RdrA with a single break between subunits and a 2.4 Å structure of RdrA with breaks between two subunits, suggesting conformational flexibility within the heptameric ring-like assembly (Figures S2D and S2E; Table S2).

AAA+ ATPase domain-containing proteins comprise a diverse family of molecular machines that hydrolyze ATP to rearrange target binding partners and mechanically funnel polypeptide and nucleic acid substrates.^18^ Similar to canonical AAA+ proteins like *Drosophila* Cdc6,^19^ the RdrA AAA+ ATPase domain contains highly conserved features associated with enzymatic activity including Walker A (G81, K82, and T83) and Walker B (D221 and D222) motifs, the sensor 1 motif (D253), and a sensor 2 motif (R377) positioned to catalyze ATP hydrolysis (Figures 2B and S2F). Although RdrA contains the core elements associated with enzymatic activity, the RdrA assembly exhibits several distinguishing features that are distinct compared with previously defined structural clades of AAA+ ATPase proteins.^20^ In contrast to most closed-ring AAA+ proteins that form hexameric assemblies, RdrA is an atypical complex with 7 repeating subunits. RdrA also lacks a pre-sensor 1 beta-hairpin insertion common in many clades of AAA+ proteins,^18^^,^^21^ and instead contains two large insertions not associated with designated AAA+ clades. The first RdrA insertion occurs after strands β2 and β3 where a canonical single helix α2 is replaced with a four-helix bundle that forms a crown at the top of the heptameric complex (discussed further in Figure 4). The second RdrA insertion occurs after the sensor 1 motif and includes three helices between strands β4 and β5 that reach into the central channel of the heptamer (Figure S2F).

**Figure S3:**
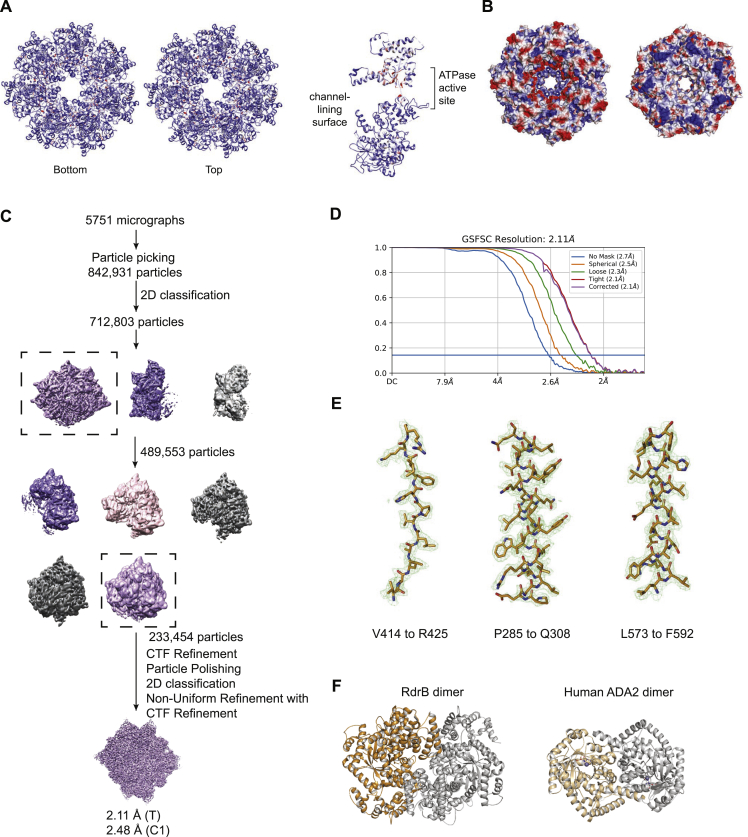
Structure of *E. coli* RdrA adenosine deaminase, related to Figure 3 (A) Amino acid conservation of RdrA among 270 RADAR systems (Table S1). Dark red indicates highly conserved amino acids and dark blue indicates no amino acid conservation. (B) Vacuum electrostatic surface display of *Ec*RdrA. (C) Particle picking and classification strategy for *E. coli* RdrB. (D) Resolution by FSC for *E. coli* RdrB. (E) Example model-to-map fit from *E. coli* RdrB with the map contoured to 5.0 σ. (F) Comparison of the dimer formed by RdrB along the 2-fold interface and the dimer formed by human ADA2.

In contrast to the N-terminal AAA+ domain, the α-helical RdrA C terminus contains no detectable homology with other known structures. To better understand the RdrA C-terminal domain, we next determined a 2.5 Å cryo-EM structure of the divergent (<20% amino acid identity) RdrA from *Streptococcus suis* (Figures 2C, S2G, and S2H; Table S2). *S. suis* RdrA adopts a similar two-layered heptameric architecture confirming conservation of the C-terminal α-helical lobe despite high sequence variability. Overall, RdrA exhibits limited amino acid conservation outside the ATPase catalytic site (Figures S3A and S3B). Interestingly, *S. suis* RdrA particles existed primarily as 14-mer complexes with the RdrA stacked head-to-tail (Figure S2G). In this conformation, although the ATPase domains are highly similar (RMSD 2.7 Å), the final four α helices of the RdrA C terminus undergo a dramatic ∼120° reorientation relative to the *E. coli* RdrA structure and flip out to form a protein-protein binding interface (Figure 2C). A notable immune protein that shares an AAA+ ATPase core structurally homologous to the RdrA ATPase is the plant NOD-like receptor ROQ1 which senses *Xanthomonas* pathogens (Figure 2D). In ROQ1-dependent immunity, sensing requires direct protein-protein interaction between the ROQ1 C-terminal leucine rich repeat domain and a *Xanthomoas* effector protein, which then induces downstream immune signaling.^22^ The C-terminal α-helical domain of RdrA may similarly act as the pathogen-sensing domain in response to an unknown phage signal.

### Structure of RdrB reveals an adenosine deaminase dodecamer

**Figure 3:**
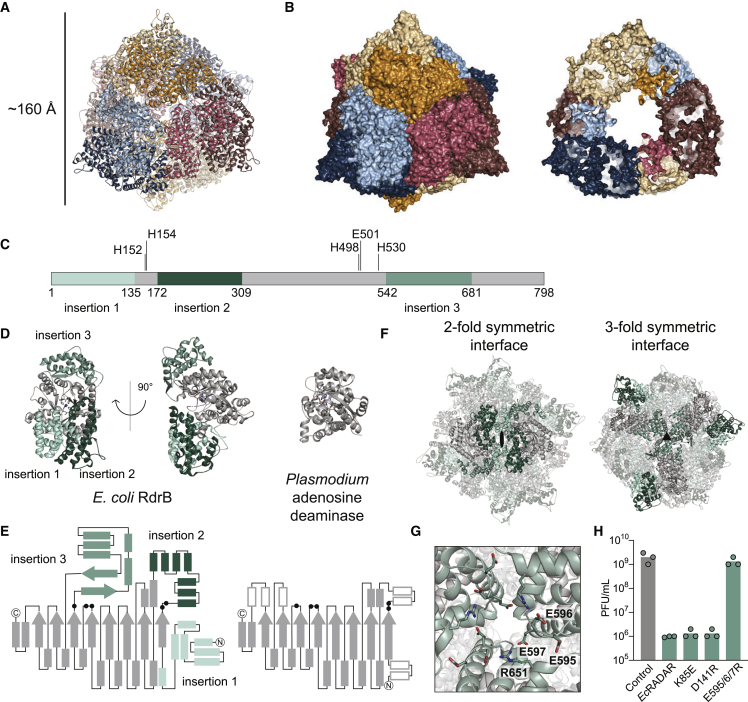
Structure of RdrB reveals an adenosine deaminase dodecamer (A) Cartoon representation of *E. coli* RdrB dodecamer, with each protomer in a different color. (B) Surface representation of *E. coli* RdrB dodecamer (left), and slice through showing hollow center (right). (C) Schematic representation showing insertions in RdrB relative to other adenosine deaminases. (D and E) Structure (D) and schematic (E) representation of single RdrB monomer highlighting insertions in shades of green (left) compared with *Plasmodium* adenosine deaminase (PDB: 2PGF) (right). Active site residues are shown as black dots in the schematic (E). (F) Insertions 1 and 2 form 2-fold symmetric dodecamer interface and insertion 3 forms the 3-fold symmetric dodecamer interface. (G) Close-up view of 3-fold interface shown in (F) highlighting key residues, including those mutated to disrupt the interface in (H). (H) Effect of point mutations suggested to disrupt the RdrB multimerization interfaces on the defensive activity of *Ec*RADAR. Data represent PFU mL^−1^ of T2 phage infecting control cells, *Ec*RADAR-expressing cells. Shown is the average of three replicates, with individual data points overlaid. The same control replicates are shown in Figures 1E and 4G for reference.

The second RADAR defense protein, RdrB, is an ∼90 kDa protein predicted to encode an adenosine deaminase domain (Figure 1A).^6^ Cryo-EM analysis of *E. coli* RdrB showed an assembly of a rigid, higher-order complex, and we determined a structure to 2.1 Å (Figures S3C–S3E; Table S2). *E. coli* RdrB forms a dodecameric assembly with 12 RdrB protomers joined to create a large hollow shell that is ∼160 Å in diameter (Figures 3A and 3B).

Structural analysis of the *E. coli* RdrB complex reveals three large insertions to the adenosine deaminase core that mediate higher order dodecameric assembly. Compared with a typical adenosine deaminase domain like *Plasmodium vivax* ADA (RMSD 2.4 Å),^23^ RdrB contains an N-terminal extension “insertion 1,” a unique “insertion 2” that divides the catalytic core, and an “insertion 3” after the final catalytic histidine H530 (Figures 3C–3E). Insertions 1 and 2 form a 2-fold symmetrical interface that joins adjacent adenosine deaminase core domains (Figure 3F). Insertion 3 forms a hydrophilic interface consisting of alternating glutamate and arginine residues that create a 3-fold symmetry axis and enable assembly of the large dodecameric shell (Figures 3F and 3G). Some adenosine deaminases are known to function as dimers (Figure S3F),^24^ but to the best of our knowledge, assembly of a large, shelled complex is a feature unique to RADAR RdrB. We made several mutations along the interfaces between RdrB protomers (Figure 3H); charge disrupting mutations to the conserved glutamate residues that line interface 3 (E595R, E596R, and E597R) result in complete loss of anti-phage defense and demonstrate that RdrB complex assembly is essential for RADAR function (Figures 3G and 3H).

### RdrA and RdrB form a supramolecular complex required for anti-phage defense

**Figure S4:**
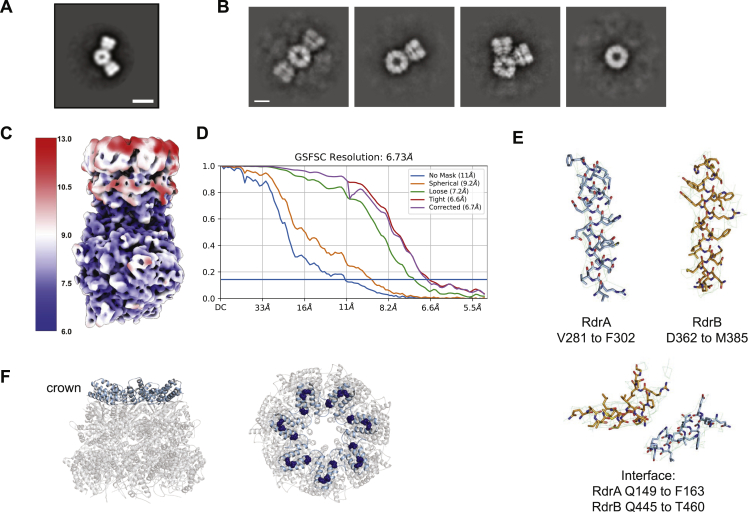
Structure of *E. coli* RdrA-RdrB supramolecular complex, related to Figure 4 (A) 2D class average from negative stain electron microscopy of RdrA-RdrB complex with a central protein surrounded by two petals. Scale bar represents 100 Å. (B) Additional 2D class averages of cryo-EM RdrA-RdrB mixture showing many arrangements with different numbers of RdrA petals surrounding a RdrB core. Scale bar represents 100 Å. (C and D) Local resolution (C) and resolution by FSC (D) for the *E. coli* RdrA-RdrB complex. (E) Example model to map fit from the *E. coli* RdrA-RdrB complex with the map contoured to 5.0 σ. (F) Cartoon of *E. coli* RdrA heptamer highlighting the crown in light blue (left), and the residues mutated to disrupt interface with RdrB (K150E, E151K, and R162E as dark blue spheres, right).

**Figure 4:**
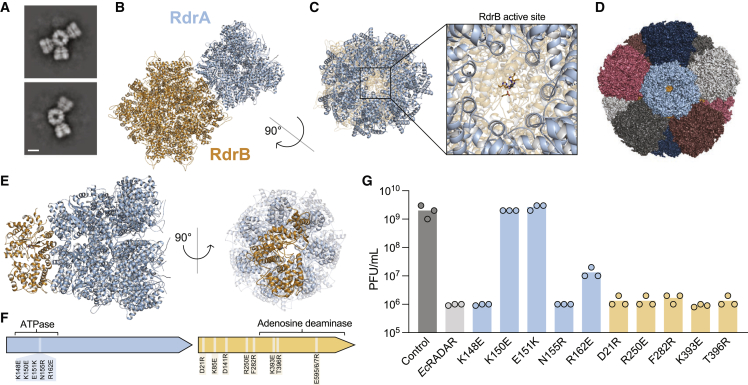
RdrA and RdrB form a supramolecular complex required for anti-phage defense (A) 2D class averages of cryo-EM RdrA-RdrB mixture showing many arrangements with different numbers of RdrA petals surrounding a RdrB core. Scale bar represents 100 Å. (B) Cartoon representation of RdrA heptamer (blue) in complex with a RdrB dodecamer (yellow). (C) View down the central channel of RdrA heptamer (blue) showing access to RdrB active site directly at the base (yellow). (D) Model of RdrB dodecamer with a RdrA heptamer bound to each of the 12 protomers, showing there is space to accommodate each RdrA heptamer in this hypothetical “saturated” complex. (E) RdrB monomer and RdrA heptamer, colored as in (D), showing the footprint of RdrA is largely restricted to a single RdrB protomer. (F) Location of mutation in RdrA and RdrB. (G) Effect of point mutations suggested to disrupt RdrA-RdrB complex on the defensive activity of *Ec*RADAR. Data represent PFU mL^−1^ of T2 phage infecting control cells. Shown is the average of three replicates, with individual data points overlaid. The same control replicates are shown in Figures 1E and 3H for reference. Scale bar represents 100 Å.

RdrA and RrdB are encoded in tandem within all known RADAR systems and each protein is essential for anti-phage defense (Figure 1).^6^ We therefore hypothesized that the two protein assemblies may directly interact. We analyzed mixed samples of purified *E. coli* RdrA and RdrB by negative-stain EM and observed gigantic flower-like arrangements with a central hub surrounded by up to six “petals” (Figure S4A). Further analysis with cryo-EM confirmed specific RdrA-RdrB co-complex formation (Figure 4A) and allowed us to determine a 6.7 Å structure of a representative single-petal complex (Figures S4B–S4E; Table S2). Placement of the high-resolution *E. coli* RADAR protein models in the RdrA-RdrB cryo-EM map reveals that the RdrB dodecamer forms the central hub and RdrA heptamers dock on the outside to create the extended petals observed by negative-stain EM (Figure 4B).

In the RdrA-RdrB complex, RdrA is positioned such that the central heptameric channel funnels directly into the catalytic core of RdrB (Figure 4C). In agreement with negative-stain EM analysis of multi-petal complexes, the RdrA heptamer footprint sits primarily over a single RdrB protomer and leaves room for a total of 12 docked RdrA complexes on the RdrB core dodecameric shell (Figures 4D and 4E). Nearly all contacts between RdrA and RdrB are mediated by residues that reside in an RdrA α-helical insertion that forms a crown on top of the AAA+ ATPase domain (Figures 2 and S4F). Modeling of a complete ∼10 MDa flower-like RdrA-RdrB complex demonstrates that the RdrB adenosine deaminase active site remains solvent-accessible, suggesting that supramolecular assembly could represent that active state of the RADAR defense complex. We introduced several mutations predicted to disrupt the formation of the RdrA-RdrB complex, including charge-swap mutations of conserved residues in the RdrA crown (K150E, E151K, and R162E) (Figures 4F and S4F) and observed that mutations to residues involved in RdrA-RdrB complex formation significantly decrease or ablate RADAR defense (Figure 4G). Together, these results reveal that supramolecular complex formation is an essential step of RADAR anti-phage defense.

### Structural analysis of RdrB suggests targeting of nucleotide substrates

**Figure S5:**
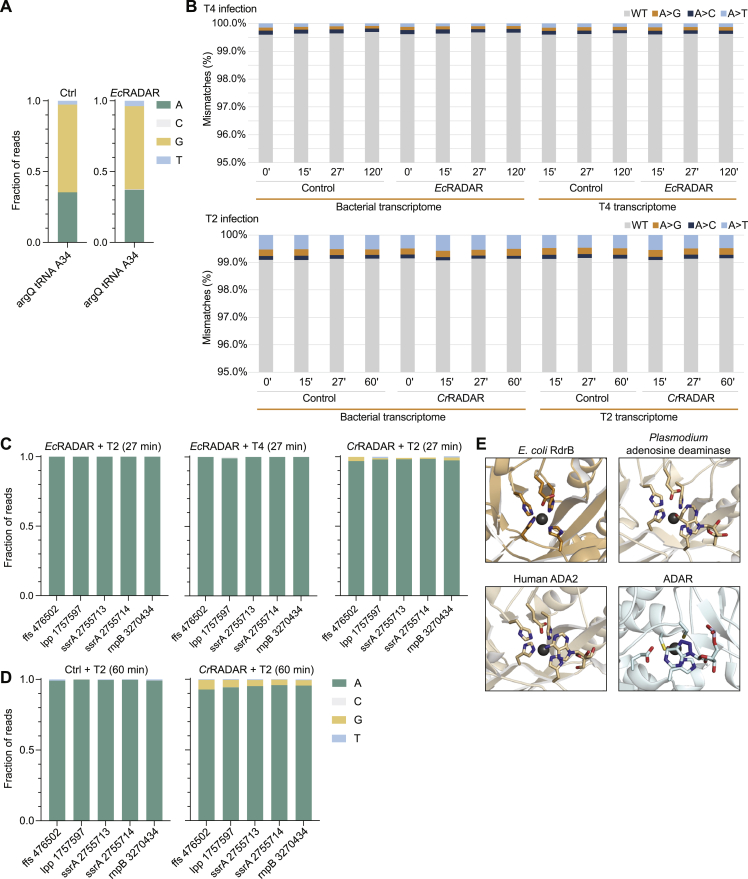
Analysis of deaminase activity of RdrB, related to Figure 5 (A) Detection of A-to-G mutational signature caused by A-to-I editing of the arginine tRNA argQ, confirming the ability of the RNA-seq pipeline to detect editing in control and RADAR-containing uninfected cells. (B) Mismatches between sequenced RNA and genomic DNA in adenine positions. RNA, extracted from *Ec*RADAR and *Cr*RADAR-expressing cells or control cells infected by phages T4 or T2 (respectively), was sequenced and the resulting reads were aligned to the reference sequences of the *E. coli* host and the respective phage genome. Shown in the rate of mismatches in all expressed adenosine positions mapped to non-rRNA genes. x axis depicts the time from the onset of infection, with t = 0 reflecting uninfected cells; y axis is the observed rate of mismatches. (C) A-to-G mutational signature in T2- and T4-infected cells with or without *Ec*RADAR and *Cr*RADAR 27 min after infection, showing select genome locations that are abundantly expressed and were previously reported to contain an A-to-G signature.^6^ (D) A-to-G mutational signature in T2-infected cells with or without *Cr*RADAR 60 min after infection, showing select genome locations that are abundantly expressed and were previously reported to contain an A-to-G signature.^6^ (E) Comparison of adenosine deaminase active sites of enzymes that modify monomeric substrates (RdrB, ADA, and ADA2) and ADAR, which modifies RNA substrates using a distinct active site and is part of the cytidine deaminase superfamily.

**Figure 5:**
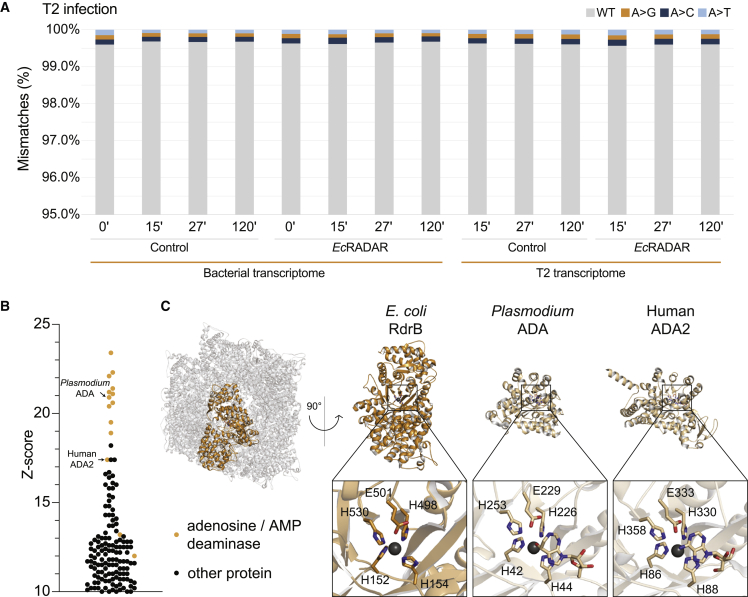
Structural analysis of RdrB suggests targeting of nucleotide substrates (A) Mismatches between sequenced RNA and genomic DNA in adenine positions. RNA, extracted from *Ec*RADAR-expressing or control cells infected by phage T2, was sequenced and the resulting reads were aligned to the reference sequences of the *E. coli* host and the T2 phage genomes. Shown in the rate of mismatches in all expressed adenosine positions mapped to non-rRNA genes. x axis depicts the time from the onset of infection, with t = 0 reflecting uninfected cells; y axis is the observed rate of mismatches. (B) DALI *Z* score of protein structures similar to RdrB, showing homology to adenosine and AMP deaminases (highlighted in yellow). (C) Cartoon representation of RdrB dodecamer, highlighting a single monomer in yellow (left), and comparison to other adenosine deaminase active sites (right).

It was previously reported that the RADAR system from *C. rodentium* edits RNAs of the host bacteria to promote abortive infection.^6^ To test whether the *E. coli* RADAR edits host RNA during infection, we performed whole-transcriptome RNA sequencing of cells infected by T2 or T4 phages, at multiplicity of infection of 2, at several time points from the onset of infection. Sequenced RNA reads with RNA-edited adenosines (A) should show A-to-G mismatches when aligned to the reference genome.^25^ To confirm our computational pipeline was able to detect A-to-G signatures indicative of editing events, we first analyzed the RNA editing event on tRNA^Arg^, which is known to be caused by the endogenous *E. coli* tRNA deaminase TadA and observed a robust A-to-G signature (Figure S5A).^26^ However, in *Ec*RADAR-expressing cells actively defending against T2 or T4 phage infection, the vast majority of adenosine residues (>99%) in expressed RNAs remained adenosines and were not converted into inosines (Figures 5A and S5B; Table S3). No strong A-to-G signatures in the bacterial nor phage transcriptomes were observed for either phage infection trial regardless of the time point examined. We repeated the experiment with *E. coli* expressing the RADAR from *C. rodentium*, infected by phage T2 but again did not observe strong signatures of A-to-G RNA editing throughout the transcriptome despite the ability of *Cr*RADAR to defend against phage T2 infection (Figure S5B; Table S3).

It was previously shown that expression of RADAR from *C. rodentium* in phage T2-infected cells results in an A-to-G signature in specific RNA loops that reside in stem-loop secondary structures of highly expressed bacterial RNAs.^6^ To further test this model, we examined the 49 positions reported by Gao et al. as heavily edited by RADAR during phage infection, expanding the analysis to several time points during infection in cells expressing either *E. coli* RADAR or *C. rodentium* RADAR. Although select locations did show a weak A-to-G signature in cells expressing *C. rodentium* RADAR (e.g., ∼4% in ssrA after 60 min) (Figure S5D), we did not observe an A-to-G signature in cells expressing *E. coli* RADAR when infected with phage T2 nor T4 (Figure S5C; Table S3). As *E. coli* RADAR provides robust protection against these two phages in the absence of an A-to-G signature, these data collectively demonstrate that RNA editing incompletely explains the mechanism of RADAR defense. We note that our experiments were performed with a different *E. coli* K-12 strain than that used by Gao et al., which may have contributed to the differences in the extent of A-to-G mutational signature that we observed in *C. rodentium* RADAR-expressing cells.

As robust RNA editing was not observed *in vivo* during RADAR anti-phage defense, we analyzed the RdrB structure to assess potential targets for deaminase activity. Comparison of *E. coli* RdrB against all structures in the Protein Data Bank (PDB) revealed that *E. coli* RdrB has no structural similarity to adenosine deaminases acting on RNA (ADAR) proteins, which are part of the cytidine deaminase superfamily of enzymes that use a mechanistically distinct active site to edit RNA (Figures 5B and S5E).^27^^,^^28^ Instead, all the top hits with structural similarity to *E. coli* RdrB (*Z* score > 20) are adenosine or AMP deaminases that deaminate the adenosine within unphosphorylated or monophosphorylated monomeric substrates, as opposed to within RNA (Figure 5B). Importantly, *E. coli* RdrB shares the same active site architecture of a histidine tetrad that coordinates a zinc ion (*E. coli* RdrB H152, H154, H498, H530) and a catalytic glutamate residue (*E. coli* RdrB E501) as known adenosine deaminases like *Plasmodium vivax* ADA (PDB: 2PGF) and the human protein ADA2 (PDB: 3LGG) (Figure 5C).^23^^,^^24^ This structural analysis suggests that RdrB enzymatic activity may target mononucleotide substrates.

### ATP-to-ITP and dATP-to-dITP conversion mediates RADAR anti-phage defense

**Figure 6:**
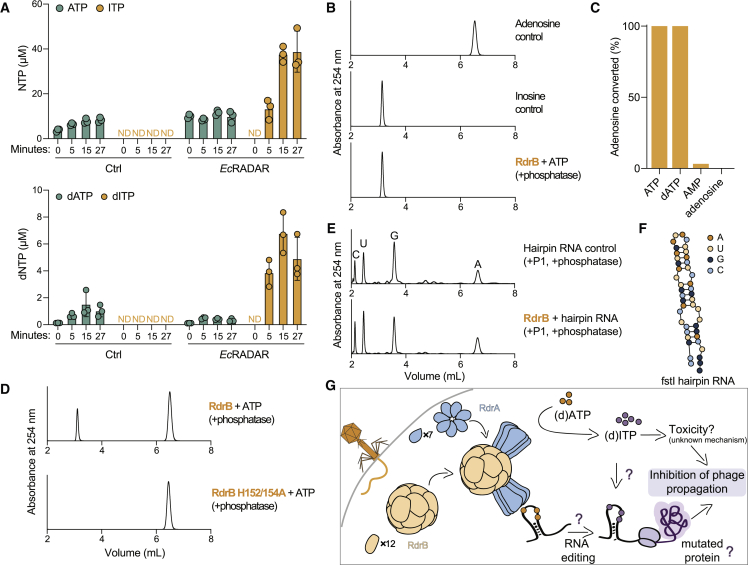
RADAR mediates ATP-to-ITP conversion in anti-phage defense (A) Quantitative mass spectrometry of ATP, dATP, ITP, and dITP in lysates extracted from cells containing *Ec*RADAR or control plasmid. Cells were infected with T4 phage at an MOI of 2 for indicated amount of time before harvesting. Bar graph shows the mean, with error bars representing standard deviation. (B) HPLC analysis of ATP after incubation with purified *E. coli* RdrB, showing deamination of ATP to ITP. (C) Summary of HPLC analyses of 1 mM different nucleotide substrates incubated with 1 μM RdrB for 30 min, demonstrating robust deamination of tri-phosphorylated substrates, but not monophosphorylated or unphosphorylated substrate. Full HPLC traces shown in Figure S6D. (D) Mutation of conserved histidine residues within the active site of RdrB are required for deaminase activity, and RdrB H152/154A mutant no longer converts ATP to ITP. (E) HPLC analysis of nucleotides after digestion of hairpin RNA to assess the identity of each base. Incubation of hairpin RNA with RdrB, in conditions where ATP is robustly converted to ITP (B), does not lead to deamination of adenosine within the RNA. (F) Schematic of fstI hairpin RNA used in (E). Hairpin was digested after incubation with RdrB, and mononucleotides were visualized by HPLC. (G) A proposed schematic for the mechanism of action of the RADAR defense system.

**Figure S6:**
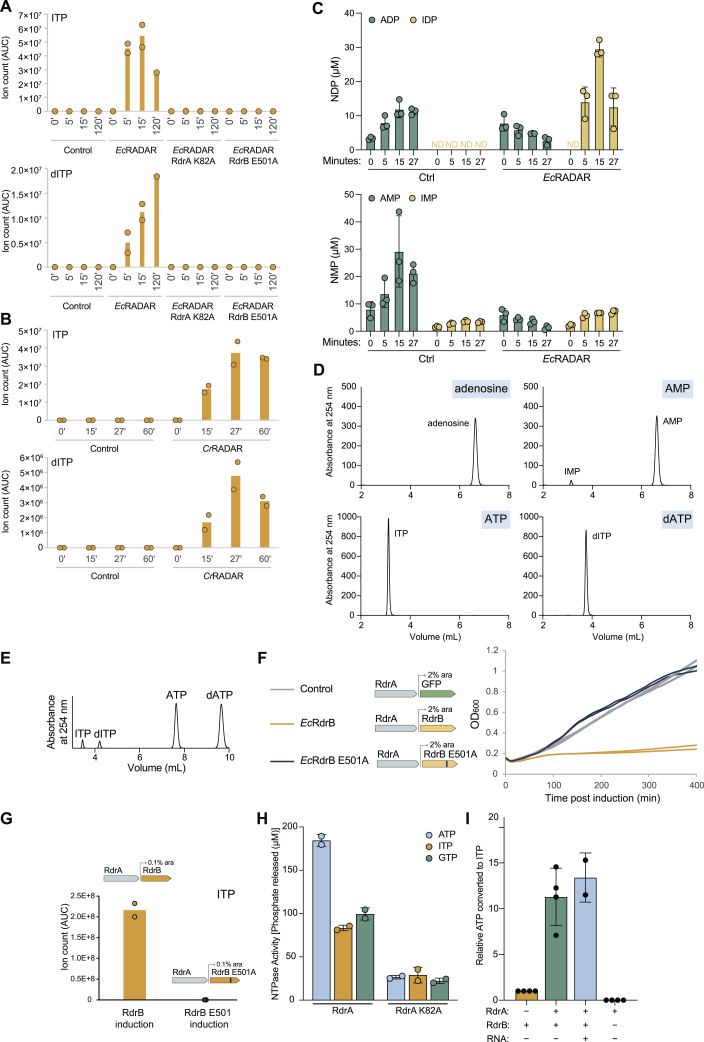
RdrB converts (d)ATP to (d)ITP *in vivo* and *in vitro*, related to Figure 6 (A) Ion count (area under curve) of ITP or dITP (respectively) in lysates extracted from WT *Ec*RADAR containing cells, as well as RADAR mutated in RdrA-K82A or RdrB-E501A, as measured by LC-MS/MS. The x axis represents min after infection, with zero representing non-infected cells. Cells were infected by phage T4 at an MOI of 2 at 37°C. Bar graphs represent the average of two biological replicates, with individual data points overlaid. (B) Ion count (area under curve) of ITP or dITP (respectively) in lysates extracted from WT *Cr*RADAR containing cells, or control cells, as measured by LC-MS/MS. The x axis represents min after infection, with zero representing non-infected cells. Cells were infected by phage T2 at an MOI of 2 at 37°C. Bar graphs represent the average of two biological replicates, with individual data points overlaid. (C) Quantitative mass spectrometry of ADP, AMP, IDP, and IMP in lysates extracted from cells containing *Ec*RADAR or control plasmid. Cells were infected with T4 phage at an MOI of 2 for indicated amount of time before harvesting. Bar graph shows the mean, with error bars representing standard deviation. (D) Full HPLC traces of data summarized in Figure 6C. (E) HPLC analysis of ATP and dATP co-incubated with RdrB, showing similar deamination of both substrates. (F) Growth curves of cells expressing *Ec*RdrB (orange), the mutant *Ec*RdrB E501A (blue), and control cells expressing GFP (gray) in the presence of 2% arabinose without phage infection. Results of two experiments are presented as individual curves. (G) Ion count (area under curve) of ITP in lysates extracted from *Ec*RdrB and EcRdrB E501A expressing cells, as measured by LC-MS/MS. Cells were supplemented with 0.1% arabinose and incubated at 37°C for 100 min. Bar graphs represent the average of two biological replicates, with individual data points overlaid. (H) Phosphate release assay measuring NTP hydrolysis by RdrA. Purified RdrA was incubated with indicated NTP, then phosphate released by hydrolysis was quantified using malachite green. Bar graph shows the mean, with error bars representing standard deviation. (I) ATP-to-ITP conversion by RdrB either alone, in the presence of RdrA, or in the presence of RdrA and hairpin RNA. RdrA alone does not deaminate ATP to ITP. Bar graph shows the mean, with error bars representing standard deviation.

As the structural analysis of RdrB pointed to homology to adenosine deaminases acting on mononucleotides, we examined the possibility that RADAR converts ATP or dATP to inosine derivatives during phage infection. We infected *E. coli* RADAR-expressing cells with phage T4 and collected cell lysates from several time points during infection. Analysis of filtered cell lysates via mass spectrometry showed accumulation of both ITP and dITP during infection, as early as 5 min from the onset of infection (Figure 6A). By 15 min after infection, cell lysates contained >3× more ITP than ATP, and >15× more dITP than dATP, highlighting the massive conversion of (d)ATP into (d)ITP that occurs in RADAR-expressing cells during infection (Figure 6A). Similar experiments with cells in which RdrA or RdrB were inactivated by point mutations in the enzyme active sites showed no ITP or dITP accumulation, demonstrating that the specific activity of both of RADAR proteins is necessary for mononucleotide conversion *in vivo* (Figure S6A). Accumulation of ITP and dITP was also observed in cells expressing RADAR from *C. rodentium* during infection by phage T2 (Figure S6B). These results show that RADAR generates substantial amounts of both ITP and dITP in response to phage infection.

Given the massive accumulation of ITP and dITP in cells expressing RADAR systems, we next tested whether RdrB could directly convert ATP to ITP *in vitro*. HPLC analysis demonstrates that purified *E. coli* RdrB is alone sufficient to catalyze rapid conversion of ATP to ITP (Figure 6B). We tested a panel of mononucleotide substrates and observed that *E. coli* RdrB robustly deaminates both ATP and dATP substrates with equal efficiency, whereas no significant activity was observed with AMP or adenosine mononucleotides (Figures 6C, S6D, and S6E). Mutations to the *E. coli* RdrB conserved histidine tetrad shared with canonical adenosine deaminase enzymes disrupted all detectable deaminase activity (Figure 6D). Finally, we tested a model dsRNA hairpin substrate reported to be a RADAR target *in vivo*^6^ and observed that under the same reaction condition sufficient for complete conversion of mononucleotides, no RNA A-to-I base-editing occurred (Figure 6E).

To better understand potential signaling between RdrA and RdrB, we next tested the impact of RdrA on RdrB activity. RdrA is an active ATPase *in vitro* that preferentially hydrolyzes ATP (Figure S6H). Compared with ATP, RdrA only weakly hydrolyzes ITP and GTP, refuting a possible model where ITP synthesized by RdrB is preferentially utilized by RdrA. Instead, we observed that the addition of RdrA to RdrB leads to enhanced conversion of ATP to ITP supporting that activation of RdrA *in vivo* likely leads to induction of RdrB activity (Figure S6I). Together, these data demonstrate that rapid deamination of adenosine nucleotides is a key mediator of RADAR anti-phage defense.

## Discussion

Our results reveal that the RADAR defense system forms a supramolecular complex that targets adenosine nucleotides and protects bacteria from phage replication. Cryo-EM structures define the mechanism of RADAR complex assembly and demonstrate that heptameric RdrA subunits dock around a core dodecameric RdrB shell to create a giant, flower-shaped defense complex (Figure 4). RdrA subunits form a funnel over the RdrB active site, and we show that the RdrB catalytic center is structurally homologous to adenosine deaminases that act on mononucleotide substrates like AMP. Upon phage infection, RADAR induces rapid accumulation of ITP and dITP. Purified RdrB catalyzes conversion of ATP to ITP and dATP to dITP *in vitro*, confirming the direct ability of RADAR to target mononucleotide substrates (Figure 6). In contrast to efficient targeting of adenosine mononucleotides, we recorded only a minor presence of A-to-G mutational signature on RNA during phage defense and could not detect RNA editing *in vitro* under conditions where ATP is robustly converted to ITP. We propose a model in which rapid accumulation of inosine derivatives poisons the nucleotide pool to inhibit phage replication and induce abortive infection (Figure 6G). In agreement with the hypothesis that accumulation of inosine derivatives is poisonous to the cell, overexpression of RdrB in the absence of phage infection results in ITP accumulation and cellular toxicity (Figures S6F and S6G). Although the massive accumulation of (d)ITP may lead to misincorporation in DNA or RNA, the precise effects of ITP accumulation in the cell after phage infection are not known. Incorporation of (d)ITP into nascent DNA or RNA may not be detectable by sequencing approaches, as the misincorporation of an inosine in place of a guanosine to base pair with a cytosine will likely show the sequence signature of a guanosine and this misincorporation would therefore be masked. Future studies are needed to understand what precisely leads to abortive infection after activation of RADAR and accumulation of (d)ITP.

The cellular nucleotide pool has emerged as a common target for host-directed immune responses that disrupt viral replication. In animal cells, SAMHD1 is a triphosphohydrolase enzyme that depletes dNTPs to limit replication of viruses including retroviruses and herpesviruses.^29^ Recently, nucleotide depletion was identified as a key effector mechanism in anti-phage defense^15^^,^^30^ and interbacterial competition.^31^ In addition to depleting available nucleotides in the cell, immune systems in animal cells and bacteria use viperin enzymes to synthesize nucleotide analogs including CTP derivatives that function as chain-terminators to disrupt replication of diverse viruses and phages.^10^^,^^32^ RADAR synthesis of inosine nucleotides provides a mechanistically similar form a defense and limits phage replication by likely increasing nucleotide misincorporation and potentially inhibiting abundant viral enzymes that require ATP hydrolysis for function, for example phage terminase proteins required for genome translocation and capsid packaging.^33^ The enormous metabolic demand required for genome synthesis creates an opportunity for the host to target free nucleotides and disrupt viral replication. We speculate that an advantage of RADAR synthesis of inosine nucleotides is the relatively low toxicity associated with spurious, low-level ATP-to-ITP conversion in the absence of infection and high antiviral inhibitory activity of elevated levels of inosine synthesis during phage replication and full system activation.

One of the most surprising findings from our structural analysis is that the RADAR components form a giant, highly unusual, multimeric assembly. Although some CRISPR immune systems form large complexes to detect invading viral nucleic acid and mount defense,^34^ the specific role of extensive multimerization of repeating subunits in RADAR defense is unknown. However, mutations that disrupt RdrA and RdrB multimerization or RdrA-RdrB complex formation inhibit defense *in vivo* and clearly demonstrate that full assembly is required for inhibition of phage replication (Figure 4). A common theme in mammalian innate immunity, including in inflammasome, Toll-like receptor, RIG-I like receptor, and cGAS-STING signaling pathways, is supramolecular complex assembly as a critical step that enhances pathogen detection and downstream immune activation.^35^ Protein oligomerization and multimerization is also required for effector activation in CBASS immunity,^36^^,^^37^^,^^38^ suggesting that RADAR complex assembly may specifically enhance enzymatic activity to enable anti-phage defense. Building on the structures of RdrA and RdrB and discovery of mononucleotide targeting, future studies will explain how RADAR complex formation regulates enzymatic function and anti-phage defense.

### Limitations of the study

Like many recently described anti-phage defense systems, it is unclear what specific molecular feature of phage infection induces activation of the RADAR system. We demonstrate that RADAR is rapidly activated upon infection, with ITP and dITP accumulating in the cell within 5 min of early phage replication. We hypothesize that the uncharacterized C-terminal domain of RdrA may act as a sensor, which upon detection of phage replication can promote RADAR complex formation and RdrB adenosine deaminase activity. Therefore, our *in vitro* experiments may lack a phage component that is necessary for activation of RADAR *in vivo*. A major focus of future experiments will be to understand what features of phage infection induce defense and to define how RdrA may convert this pathogen recognition event into RADAR complex activation.

## STAR★Methods

### Key resources table

**Table:** 

REAGENT or RESOURCE	SOURCE	IDENTIFIER
**Bacterial and virus strains**		

*E. coli* BL21-DE3 RIL	Agilent	230245
*E. coli* MG1655	Coli Genetic Stock Center	CGSC6300
Phage T2	German Collection of Microorganisms and Cell Cultures GmbH (DSMZ)	DSM 16352,Accession: LC348380.1
Phage T4	U. Qimron	Accession: AF158101.6
Phage T6	German Collection of Microorganisms and Cell Cultures GmbH (DSMZ)	DSM 4622,Accession: MH550421.1
Phage T5	U. Qimron	Accession: AY543070.1
Phage T7	U. Qimron	Accession: NC_001604.1
Lambda vir	U. Qimron	Accession: NC_001416.1

**Chemicals, peptides, and recombinant proteins**		

Ni-NTA Agarose	Qiagen	30250
HiTrap Q HP Column	Cytvia	17115401
Zorbax Bonus-RP	Agilent	863668-901
SRT SEC-300	Sepax	215300-7830
Nuclease P1 from *Penicillium citrinum*	Sigma-Aldrich	N8630
Alkaline Phosphatase, Quick CIP	New England Biolabs	M0525S
Quantifoil R 2/1 300 mesh grids, copper	Electron Microscopy Sciences	FCF400-Cu

**Recombinant DNA**		

pBbE8k-RFP	Lee et al.^39^	Addgene Cat#35276
pSG1	Doron et al.^5^	N/A
pBAD-GFP	Thermo Fisher Scientific	Cat #43001
*E. coli* P0304799.3 RADAR	Genscript Corp.	N/A
*C. rodentium* DBS100 RADAR	Genscript Corp.	N/A
*S. suis* SS993 RADAR	Genscript Corp.	N/A
*Ec*RADAR_A_K82A	Genscript Corp.	N/A
*Ec*RADAR_A_E151K	Genscript Corp.	N/A
*Ec*RADAR_A_N155R	Genscript Corp.	N/A
*Ec*RADAR_A_K148E	Genscript Corp.	N/A
*Ec*RADAR_A_K150E	Genscript Corp.	N/A
*Ec*RADAR_A_R162E	Genscript Corp.	N/A
*Ec*RADAR_B_E501A	Genscript Corp.	N/A
*Ec*RADAR_B_K85E	Genscript Corp.	N/A
*Ec*RADAR_B_E595/596/597R	Genscript Corp.	N/A
*Ec*RADAR_B_D141R	Genscript Corp.	N/A
*Ec*RADAR_B_K393E	Genscript Corp.	N/A
*Ec*RADAR_B_R250E	Genscript Corp.	N/A
*Ec*RADAR_B_F282R	Genscript Corp.	N/A
*Ec*RADAR_B_D21R	Genscript Corp.	N/A
*Ec*RADAR_B_T396R	Genscript Corp.	N/A

**Deposited data**		

*Ec*RdrA unsplit	This paper	EMD: 29323, PDB: 8FNT
*Ec*RdrA single split	This paper	EMD: 29324
*Ec*RdrA double split	This paper	EMD: 29325
*Ss*RdrA	This paper	EMD: 29326, PDB: 8FNU
*Ec*RdrB	This paper	EMD: 29327, PDB: 8FNV
*Ec*RdrA–*Ec*RdrB	This paper	EMD: 29328, PDB: 8FNW

**Oligonucleotides**		

Primers, see Table S5	This paper	N/A

**Software and algorithms**		

Phenix 1.13-2998	Adams et al.^40^	https://www.phenix-online.org/
Coot 0.8.9	Emsley and Cowtan^41^	https://www2.mrc-lmb.cam.ac.uk/personal/pemsley/coot/
Pymol v1.7.4.4	Schrödinger, LLC	https://pymol.org/
Prism 7.0d	GraphPad software	https://www.graphpad.com/scientific-software/prism/

### Resource availability

#### Lead contact

Further information and requests for resources and reagents should be directed to and will be fulfilled by the lead contact, Philip Kranzusch (philip_kranzusch@dfci.harvard.edu).

#### Materials availability

This study did not generate new unique reagents.

### Experimental model and subject details

#### Bacterial strains and phages

*E. coli* strain MG1655 (ATCC 47076) was grown in MMB (LB supplemented with 0.1 mM MnCl_2_, 5 mM MgCl_2_, with or without 0.5% agar) at 37°C or room temperature (RT). Whenever applicable, media were supplemented with ampicillin (100 μg mL^−1^), to ensure the maintenance of plasmids. Infection was performed in MMB media at 37°C or RT as detailed in each section. Phages used in this study are listed in the key resources table.

### Method details

#### Protein expression and purification

Recombinant *E. coli* RdrA and RdrB and *S. suis* RdrA were purified using methods previously described.^36^ Briefly, RdrA and RdrB were cloned into an N-terminal 6×His-SUMO2-tagged pET vector and transformed into BL21-RIL *E. coli* (Agilent). Large scale cultures (2–4 liters) were grown for ∼5 h at 37°C, then induced with IPTG overnight at 16°C. Bacterial pellets were resuspended and sonicated in lysis buffer (20 mM HEPES-KOH pH 7.5, 400 mM NaCl, 30 mM imidazole, 10% glycerol and 1 mM DTT) and purified using Ni-NTA resin (Qiagen). Ni-NTA resin was washed with lysis buffer supplemented to 1 M NaCl and eluted with lysis buffer supplemented to 300 mM imidazole. The Ni-NTA elution fraction was dialyzed into 20 mM HEPES-KOH pH 7.5, 250 mM KCl, 1 mM DTT overnight while removing the SUMO2 tag with recombinant human SENP2 protease (D364–L589, M497A). RdrA was bound to a Q column (Cytvia) and eluted with a gradient of KCl from 150 mM to 1 M. RdrA and RdrB were each concentrated using a 30K-cutoff concentrator (Millipore) and purified by size exclusion chromatography on a 16/60 Sephacryl 300 column. Proteins were concentrated to >10 mg mL^−1^, flash frozen with liquid nitrogen, and stored at −80°C.

#### Plasmid and strain construction

RADAR operons used for phage challenge assays in this study were synthesized by Genscript Corp. and cloned into the p15a-origin-containing pSG1 plasmid with their native promoters, or into the pBAD plasmid (Thermofisher, cat. #43001), as previously described.^10^ Mutants of the system were also synthesized and cloned by Genscript. All synthesized sequences are presented in Table S4. Inducible mutants of RdrB were constructed using Q5 Site directed Mutagenesis kit (NEB, cat. #E0554S), using primers described in Table S5.

#### Plaque assays

Phages were propagated by picking a single phage plaque into a liquid culture of *E. coli* MG1655 grown at 37°C to OD_600_ of 0.3 in MMB medium until culture collapse. The culture was then centrifuged for 10 min at 15,000 × g and the supernatant was filtered through a 0.2 μm filter to get rid of remaining bacteria and bacterial debris. Lysate titer was determined using the small drop plaque assay method as described before.^42^

Plaque assays were performed as previously described.^42^ Bacteria (*E. coli* MG1655 with pSG1 or pBAD plasmid) and negative control (*E. coli* MG1655 with empty pSG1 or pBad-GFP) were grown overnight at 37°C. Then 300 μL of the bacterial culture was mixed with 30 mL melted MMB agar (LB supplemented with 0.1 mM MnCl_2_, 5 mM MgCl_2_, 0.5% agar, with or without 0.2% arabinose) and left to dry for 1 h at room temperature. 10-fold serial dilutions in MMB were performed for each of the tested phages and 10 μL drops were put on the bacterial layer. Plates were incubated overnight at RT. Plaque forming units (PFUs) were determined by counting the derived plaques after overnight incubation.

#### Liquid toxicity assay

Overnight cultures of bacteria harboring a pSG1 plasmid with *Ec*RdrA and an inducible plasmid (pBbE8k) with different versions of *Ec*RdrB or a GFP control were diluted 1:100 in MMB medium. Cells were incubated at 37°C while shaking at 200 rpm for 1 h. 180 μL of the bacterial culture were transferred into wells in a 96-well plate supplemented with 2% arabinose and incubated at 37°C with shaking in a TECAN Infinite200 plate reader. OD_600_ was followed with measurement every 10 min.

#### Cryo-electron microscopy data collection

Solutions of purified RdrA, RdrB, and a combination of both, were applied to glow discharged grids and vitrified in liquid ethane. For RdrA and RdrB, concentrations of 2.40 and 2.35 mg mL^−1^ were used respectively. For the combination of both, RdrA and RdrB were mixed in a 1:1 ration resulting in final concentrations of 1 mg mL^−1^ and 0.98 mg mL^−1^ respectively. The ideal concentrations to use were based off the preliminary negative stain data. To vitrify the sample on TEM grids, a Mark IV Vitrobot (ThermoFisher) was used. A 3 μL solution of each sample were independently deposited onto 1.2 / 1.3 Au Quantifoil grid with Carbon mesh then blotted with filter paper for 6 seconds, using a double-sided blot with a force of 5, in a 100% relative humidity chamber at 4°C.

*S. suis* RdrA, as well as *E. coli* RdrA and RdrB combination grids, were screened and imaged using a Talos Arctica (ThermoFisher) microscope operating at 200 kV and equipped with K3 direct electron detector (Gatan). Approximately 300 and 240 movies were acquired of the RdrA and the RdrA and RdrB combination sample, respectively, using SerialEM software version 3.8.6 at a pixel size of 1.1 Å, a total dose of 42.02 e− /Å^2^, dose per frame of 1.05 e− /Å^2^. A defocus range of −0.7 to −2.0 μm was used for *E. coli* RdrA and RdrB combination grids while a defocus range of −0.5 to −3.0 μm was used for *S. suis* RdrA.

*E. coli* RdrA and *E. coli* RdrB grids were screened and imaged using a Titan Krios microscope operating at 300 kV and equipped with a K3 direct electron detector with energy filter (Gatan). All data was acquired using SerialEM software version 3.8.6 at a pixel size of 0.825 Å and a defocus range of −0.5 to −2.5 μm. Approximately 11,200 movies of *E. coli* RdrA were collected at a total dose of 48.8 e− /Å^2^, dose per frame of 1.16 e− /Å^2^. Approximately 5,700 movies of *E. coli* RdrB were collected with a total dose of 48.8 e− /Å^2^, dose per frame of 1.16 e− /Å^2^.

#### Cryo-EM data processing

Dose-fractionated images of *E. coli* RdrA were gain normalized and motion corrected with MotionCor2 (v1.3.1)^43^ followed by CTF and defocus value determination in CTFFIND4.^44^ Particle picking was carried out in crYOLO^45^ resulting in 2,296,605 initial particles. Following multiple rounds of 2D classification in RELION^46^ to remove erroneous picks, contamination, and “junk” particles 1,578,051 particles representing intact RdrA were obtained. Heterogeneous refinement in cryosparc^47^ identified three different populations of RdrA, totaling 1,314,627 particles. Following particle polishing^48^ and CTF refinement^49^ in RELION on the combined data a further cycle of heterogeneous refinement followed by Non-uniform refinement^50^ and additional CTF refinement in cryosparc resulted in the “no split”, “single split”, and “double split” reconstructions at resolutions of 2.3, 2,5 and 2.5 Å respectively (Figure S2A). *E. coli* RdrB was processed in a similar manner with 842,931 particles initial identified, leading to 712,803 after 2D classification. Following multiple rounds of heterogeneous refinement in cryopsarc 233,454 particles were subjected to polishing, CTF refinement, particle polishing, additional 2D classification and finally Non-uniform refinement resulting in a 2.1 Å reconstruction with T symmetry (2.5 Å C1) (Figure S3A).

The small set of *E. coli* RdrA-RdrB complex images were motion corrected using the RELION implementation, followed by CTFFIND4 and particle picking in crYOLO, resulting in 20,409 particles. All particles were subjected to ab initio reconstruction into three classes in cryosparc. 9,236 particles were identified corresponding to the complex, resulting in a 6.7 Å reconstruction following refinement.

Dose-fractionated images of *S. suis* RdrA were gain normalized and motion corrected with MotionCor2 (v1.4.0) followed by CTF and defocus value determination in CTFFIND4.^44^ crYOLO models were trained to identify potential “monomer” (single heptametic ring) and “dimer” (double stacked rings) species. These were then combined and duplicate particles removed prior to 2D classification in RELION which resulted in 573,499 particles after “junk” removal. These particles then underwent multiple rounds of heterogeneous refinement within cryosparc alongside CTF refinement resulting in 193,305 particles in total. These particles were polished in RELION before a final round of CTF refinement and Non-uniform refinement in cryosparc resulting in the final C7 reconstruction at 2.5 Å (2.7 Å C1).

Structural biology applications other than cryosparc used in this project were compiled and configured by SBGrid.^51^

#### Negative stain electron microscopy

*E coli* RdrA and RdrB were mixed to a final concentration of 50 nM for each protein in buffer containing 100 mM KCl, 50 mM HEPES pH 7.5, and 1 mM TCEP. 4 μL samples were applied to glow-discharged copper grids (Electron Microscopy Sciences, cat. #FCF400-Cu), stained with 2% uranyl formate, and imaged on a JEOL-1400 at 80kV. To determine sample quality for subsequent cryo-EM analysis, 30 micrographs were acquired at 40kx magnification (3.3 pixels nm^-1^) and used for 2D classification in RELION.^46^ Select 2D classes representing different arrangements of RdrA petals around the RdrB core were analyzed in ImageJ to add scale bars.

#### RNA sequencing

Overnight cultures of bacteria (*E. coli* MG1655 harboring pSG1-*Ec*RADAR or pSG1-*Cr*RADAR plasmid) or negative control (*E. coli* MG1655 with the pSG1 plasmid) were diluted 1:100 in 60 mL of MMB medium and incubated at 37°C while shaking at 200 rpm until early log phase (OD_600_ of 0.3). 10 mL samples of each bacterial culture were taken and centrifuged at 4000 rpm for 5 min at 4°C. The pellets were flash frozen using dry ice and ethanol. The remaining cultures were infected by phage T4 or T2, at a final MOI of 2. 10 mL samples were taken throughout infection at 0, 15, 27 and 120 min post infection (for *Ec*RADAR), or 0, 15, 27 and 60 min post infection (for *Cr*RADAR), and centrifuged and flash frozen as described above. RNA extraction was performed as described previously.^52^ Briefly, frozen pellets were re-suspended in 1 mL of RNA protect solution (FastPrep) and lysed by Fastprep homogenizer (MP Biomedicals). RNA was extracted using the FastRNA PRO blue kit (MP Biomedicals, 116025050) according to the manufacturer’s instructions. DNase treatment was performed using the Turbo DNA free kit (Life Technologies, AM2238). RNA was subsequently fragmented using fragmentation buffer (Ambion-Invitrogen, cat. #10136824) at 72°C for 1 min and 45 s. The reactions were cleaned by adding ×2.5 SPRI beads (Agencourt AMPure XP, Beckman Coulter, A63881). The beads were washed twice with 80% ethanol and air dried for 5 min. The RNA was eluted using water. Ribosomal RNA was depleted by using the Ribo-Zero rRNA Removal Kit (Epicentre, MRZB12424). Strand-specific RNA-seq was performed using the NEBNext Ultra Directional RNA Library Prep Kit (NEB, E7420) with the following adjustments: all cleanup stages were performed using ×1.8 SPRI beads, and only one cleanup step was performed after the end repair step. Following sequencing on an Illumina NextSeq500, sequenced reads were demultiplexed and adapters were trimmed using ‘fastx clipper’ software with default parameters. Reads were mapped to the bacterial and phage genomes by using NovoAlign (Novocraft) v3.02.02 with default parameters as previously described.^52^ Reads mapped to rRNA genes were discarded. Reads mapping equally well to multiple positions in the reference genome, as well as reads containing insertions and deletions as compared to the reference genome, were also discarded. Only reads mapping to the antisense strand of annotated genes were used for the mutation analyses, as these reads represent cDNA generated from the mRNA. Mutations from reference genomes were identified and quantified by counting each mismatch across the transcriptome. Frequency of mismatches was compared between control and RADAR samples throughout the infection time course.

#### Cell lysate preparation

Overnight cultures of *E. coli* harboring the defensive system and negative controls were diluted 1:100 in 250 mL MMB medium (with or without 0.2% arabinose, as described in Table S4 and grown at 37°C (250 rpm) until reaching OD_600_ of 0.3. The cultures were infected by T2 or T4 at a final MOI of 2. Following the addition of phage, at 5, 15 and 60 or 120 min post infection (plus an uninfected control sample), 50 mL samples were taken and centrifuged for 5 min at 15,000 × g. Pellets were flash frozen using dry ice and ethanol. The pellets were re-suspended in 600 μL of 100 mM phosphate buffer at pH 8 and supplemented with 4 mg mL^−1^ lysozyme. The samples were then transferred to a FastPrep Lysing Matrix B 2 mL tube (MP Biomedicals cat. #116911100) and lysed using a FastPrep bead beater for 40 s at 6 m s^−1^ (two cycles). Tubes were then centrifuged at 4°C for 15 min at 15,000 × g. Supernatant was transferred to Amicon Ultra-0.5 Centrifugal Filter Unit 3 kDa (Merck Millipore cat. #UFC500396) and centrifuged for 45 min at 4°C at 12,000 × g. Filtrate was taken and used for LC-MS analysis.

#### Detection of inosine compounds by untargeted HPLC-MS

Profiling of polar metabolites was done as previously described^53^ with minor modifications as described below. In brief, the analysis was performed using an Acquity I class UPLC System combined with a mass spectrometer (Thermo Exactive Plus Orbitrap), which was operated in a positive ionization mode using a mass range of 200–800 m/z. The LC separation was done using the SeQuant Zic-pHilic (150 mm × 2.1 mm) with the SeQuant guard column (20 mm × 2.1 mm) (Merck). The mobile phase B was acetonitrile and mobile phase A was 20 mM ammonium carbonate plus 0.1% ammonia hydroxide in water. The flow rate was kept at 200 μl min^−1^ and the gradient was as follows: 75% B (0–2 min), decrease to 25% B (2–14 min), 25% B (14–18 min), increase back to 75% B (18–19 min), 75% B (19–23 min). Inosine derivatives peaks were identified in the data using MSMS fragmentation, by identifying the inosine base signature as well as phosphates, ribose or deoxyribose. Area under the peak was quantified using MZmine 2.53^54^ with an accepted deviation of 5 ppm.

#### Quantitative MS methods

Cell lysates were prepared as described above and sent for analysis at the Targeted Metabolomics unit of the Weizmann institute. Quantification of nucleotides was carried out using an Acquity I-class UPLC system coupled to Xevo TQ-S triple quadrupole mass spectrometer (both Waters). The UPLC was performed using a SeQuant ZIC-pHILIC column as described previously,^55^ with linear gradient decrease of acetonitrile in 20 mmol L^−1^ ammonium carbonate for 10 min. Mass spectrometry analysis was performed using electrospray interface in positive ionization mode for all metabolites. Metabolites were detected using multiple-reaction monitoring (MRM), using argon as the collision gas. Quantification was made using a standard curve in the 0–1 μg mL^−1^ concentration range. ^13^C_10_-ATP and ^15^N_5_-AMP were added to standards and samples as internal standards to get final concentrations 10 μmol L^−1^ and 0.5 μmol L^−1^, respectively. TargetLynx (Waters) was used for data analysis. Standards and materials used for this analysis were purchased from the following resources. Deoxyadenosine 5′-monophosphate (dAMP), deoxyadenosine (dA), deoxyinosine (dI), inosine 5′-diphosphate (IDP), inosine (I), ^13^C_10_-adenosine 5’-triphosphate (^13^C_10_-ATP) and ^15^N_5_-adenosine 5′-monophosphate (^15^N_5_-AMP) were purchased from Merck. Deoxyinosine 5’-triphosphate (dITP), deoxyinosine 5’-monophosphate (dIMP), and Inosine 5′-triphosphate (ITP) were purchased from Santa Cruz Biotechnology. Deoxyadenosine 5′-diphosphate (dADP) was purchased from Alfa Aesar.

#### Analysis of base editing by HPLC

All RdrB reactions were carried out at 37°C in standard reaction conditions: 50 mM KCl, 50 mM HEPES pH 7.5, 1 mM TCEP, 10 mM MgCl_2_, 100 μM ZnSO_4_, and 1–10 μM *E. coli* RdrB. For monomeric substrates (ATP, dATP, AMP, adenosine), 1 mM final concentration of substrate was used, and reactions were incubated for 30 min before heating to 90°C to end reactions. For comparison of ATP and dATP conversion, reactions containing 1 mM ATP and 1 mM dATP were incubated at 37°C for 10 min. Hairpin RNA experiments contained 1 μM hairpin RNA and were at 37°C for 1 h. Reactions were treated with calf intestinal phosphatase (New England Biolabs) and hairpin RNA samples were concurrently treated with P1 nuclease to release monomeric NMPs for analysis, then samples were spun through a 0.2 μm filter. Analysis was carried out using a C18 column (Agilent Zorbax Bonus-RP 4.6×150 mm, 3.5-micron). The column was heated to 40°C and run at 1 mL min^−1^ with a mobile phase of 50 mM NaH_2_PO_4_ (pH 6.8 with NaOH) supplemented with 3% acetonitrile.

#### ATPase activity

Purified *E. coli* RdrA (5 μM) was incubated with 1 mM indicated NTP for 2 h with 50 mM KCl, 50 mM HEPES-KOH pH 7.5, 1 mM TCEP, and 10 mM MgCl_2_. Malachite green (Sigma-Aldrich MAK307) was used to quantify phosphate released by NTP hydrolysis, following the manufacturer’s instructions. Samples were diluted 1:8 so released phosphate was within linear range of detection based on phosphate standards. Absorbance at 650 nm of a “blank” sample with no protein was subtracted from all samples, then the absorbance was used to calculate the molarity of phosphate released based on a standard curve created using phosphate standards. Samples were measured in technical duplicate and are representative of 4 independent biological replicates.

### Quantification and statistical analysis

Statistical details for each experiment can be found in the figure legends and outlined in the corresponding method details section. Bar graphs show the average of replicates with individual points overlaid, unless stated otherwise.

## Data Availability

• Coordinates of the structures *E. coli* and *S. suis* RdrA, *E. coli* RdrB, and *E. coli* RdrA-RdrB complex have been deposited under the following accession numbers: 8FNT, 8FNU, 8FNV, 8FNW. Maps of *E. coli* RdrA (no split, single-split, and double-split), *S. suis* RdrA, *E. coli* RdrB, and *E. coli* RdrA-RdrB complex have been deposited under the following accession numbers: EMD-29323, EMD-29324, EMD-29325, EMD-29326, EMD-29327, and EMD-29328. These data are publicly available as of the date of publication. • This paper does not report original code. • Any additional information required to reanalyze the data reported in this paper is available from the lead contact upon request.

## Supplemental information

Table S1. RADAR defense system distribution, related to Figure 1

Table S2. Structure statistics, related to Figures 2, 3, and 4

Table S3. RNA sequencing, related to Figure 5

Table S4. Sequences synthesized in this study, related to Figure 1

Table S5. Primers used in this study, related to Figure 1
